# Comparing Transcription Rate and mRNA Abundance as Parameters for Biochemical Pathway and Network Analysis

**DOI:** 10.1371/journal.pone.0009908

**Published:** 2010-03-26

**Authors:** Brewster Hayles, Sailu Yellaboina, Degeng Wang

**Affiliations:** Department of Cell Biology, Microbiology, and Molecular Biology (CMMB), University of South Florida, Tampa, Florida, United States of America; King Abdullah University of Science and Technology, Saudi Arabia

## Abstract

The cells adapt to extra- and intra-cellular signals by dynamic orchestration of activities of pathways in the biochemical networks. Dynamic control of the gene expression process represents a major mechanism for pathway activity regulation. Gene expression has thus been routinely measured, most frequently at steady-state mRNA abundance level using micro-array technology. The results are widely used in statistical inference of the structures of underlying biochemical networks, with the assumption that functionally related genes exhibit similar dynamic profiles. Steady-state mRNA abundance, however, is a composite of two factors: transcription rate and mRNA degradation rate. The question being asked here is therefore whether steady-state mRNA abundance or any of two factors is a more informative measurement target for studying network dynamics. The yeast *S. cerevisiae* was used as model organism and transcription rate was chosen out of the two factors in this study, because genome-wide determination of transcription rates has been reported for several physiological processes in this species. Our strategy is to test which one is a better measurement of functional relatedness between genes. The analysis was performed on those *S. cerevisiae* genes that have bacterial orthologs as identified by reciprocal BLAST analysis, so that functional relatedness of a gene pair can be measured by the frequency at which their bacterial orthologs co-occur in the same operon in the collection of bacterial genomes. It is found that transcription rate data is generally a better parameter for functional relatedness than steady state mRNA abundance, suggesting transcription rate data is more informative to use in deciphering the logics used by the cells in dynamic regulation of biochemical network behaviors. The significance of this finding for network and systems biology, as well as biomedical research in general, is discussed.

## Introduction

Biochemical networks underlie essentially all cellular functions. Proteins encoded in the genomic sequences form biochemical pathways and pathways join together to form networks. Cellular biochemical networks are highly modular in that they consist of a hierarchical organization of functional modules [1]. For instance, metabolic enzymes form the metabolic network; protein kinases are backbones of the signaling network; and transcription factors are major components of the transcription regulation network.

The cells constantly adapt to changes in their environment and internal conditions. Consequently, biochemical networks are never static. The cells respond to extra- and intra-cellular signals by dynamic orchestration of activities of pathways in the networks; activate pathways that are needed and inactivate the others [2]. For example, during carbon source shift from glucose to galactose, the yeast cells activate galactose utilization pathways, while inactivating pathways required for glucose metabolism [3], [4].

Roughly two factors determine how active a pathway is: the abundance and the activity of its components. The activity of many proteins, the latter of the two factors, is under tight post-translational control. There are many forms of post-translational control: covalent chemical modification such as phosphorylation [5] and glycosylation [6], allosteric interaction with regulatory partners [7], etc.

The other determining factor of biochemical pathway activity, the abundance of proteins, is also under tight cellular regulation. Gene expression regulation is one of the major regulatory mechanisms. Hence, gene expression has been, and will continue to be, extensively studied. Perhaps due to the availability of reliable and economical measurement methods such as the SAGE [8], the micro-array and the upcoming deep sequencing technologies [9], mRNA transcript levels have been the most frequently studied, even though gene expression process is a multi-stepped process. Measurement targets are usually steady state mRNA abundance. The results are widely used in statistical inference of the structures of underlying biochemical networks [10], as well as in regulatory sequence analysis to detect common sequence motifs that drive transcription of functionally related genes [11], [12]. The underlying assumption is that functionally related genes should exhibit similar dynamic transcription regulation profiles. Steady-state mRNA abundance, however, is determined by two factors: transcription rate and mRNA degradation rate; and discrepancies between steady-state mRNA abundance and transcription rate have been frequently reported [3]. This raises the issue whether steady-state mRNA abundance is the best, and a sufficient, measurement of functional relatedness.

Functional relatedness is an important parameter for the construction and dynamic analysis of biochemical networks. To some degree, genes are organized into distinct, though often overlapping, functional groups. Transcription of genes in a group are activated and inactivated together. This observation is captured in the “regulon” concept to describe the strategy used by the cells to orchestrate the activity of the collection of its biochemical pathways [13]. In bacteria, a poly-cistronic operon can be considered as a regulon, as proteins belong to the same pathway are often organized into one operon [14]. Additionally, for genes that are highly functionally related, operons in which they co-occur tend to be conserved across bacterial genomes [15], [16]. Regulons or functional relatedness of a pair of genes, however, is difficult to identify in eukaryotes. Genes in eukaryotes are independent transcription units. Computational analysis of regulatory sequences to predict similar expression profiles, and functional relatedness, between a pair of genes remains technically un-practical. On the other hand, many eukaryote genes, at least in the yeast *S. cerevisiae*, have orthologs in bacteria genomes. Occurrence in the same operon by their bacteria orthologs has been shown to be a good indicator of functional relatedness for a pair of such genes in *S. cerevisiae* [13].

Moreover, genome-wide determination of transcription rates have been reported for several physiological processes in the yeast *S. cerevisiae* [3], [17], [18]. Here, we report our efforts to take advantage of these datasets to compare transcription rate and steady-state mRNA abundance as measurements of functional relatedness. Our analysis was performed on a group of *S. cerevisiae* genes that have bacterial orthologs as identified by reciprocal BLAST analysis. Functional relatedness of a gene pair is measured by the frequency at which their bacterial orthologs occur in the same operon in the collection of NCBI bacterial genomes.

## Materials and Methods

### A. Genome data and prediction of *Saccharomyces cerevisiae* orthologs in bacteria

The proteomic sequences, protein genomic locations, and complete genomic sequences of 765 bacteria species and the yeast *Saccharomyces cerevisiae* were downloaded from NCBI ftp site (ftp://ftp.ncbi.nih.gov/genomes/). Proteomes of the plasmids in all these species were not included in our analysis. In order to identify protein orthology between *S. cerevisiae* proteomes and the collection of bacterial proteomes, bi-directional BLASTp analysis was performed. Briefly, each of annotated open reading frames (ORFs) of *S. cerevisiae* was used as a query to BLAST against each of the bacterial proteomes, with 10^−4^ as the cut-off BLAST E-value [19]. Conversely, each protein sequence in a bacterial proteomes was used as a query in BLAST against *S. cerevisiae* proteome, with 10^−4^ as the cut-off E-value. Orthology between a *S. cerevisiae* protein and a bacterial protein was identified only when the pair is the best hit in both direction of the BLAST analysis. Additionally, redundancy exists in the bacterial proteome collection; many of the 765 bacterial proteomes are highly homologous, sometimes almost identical, to each other. It is known that such genome redundancy adversely affect computational prediction of functional relatedness between genes [20], [21]. Therefore, in these cases, we selected only the one that shared the maximum number of orthologs with *S. cerevisiae* to reduce redundancy. Finally, we identified a total of 284 non-redundant bacterial species that share orthologs with *S. cerevisiae*. A total of 2030 *S. cerevisiae* genes were identified to have bacterial orthologs.

### B. Bacterial operon prediction and co-operon frequency calculation

A support vector machine (SVM), as described previously [22], was used. Briefly, experimentally proven operons downloaded from the EcoCyc database were used to create a set of gene pairs, in which the genes in a pair are adjacent to each other and belong to the same operon. Additionally, we collected a set of gene pairs, in which the genes in a pair are adjacent to each other, transcribed in the same direction, but belong to different operons. Intergenic distances between the genes in the former gene pairs were taken as positive data set, whereas those in the latter were taken as the negative data set. The two datasets were then used to train a Support Vector Machine to predict poly-cistronic operons in the 284 species of bacteria. For any pair of *S. cerevisiae* genes, both of which have bacterial orthologs, we counted how many times their orthologs occurred in the same operon — the co-operon frequency. Using this method, we identified 8636 gene pairs, among 1491 *S. cerevisiae* genes, to have their bacterial orthologs co-occur at least once in predicted operons.

The co-operon frequency dataset generated above was further processed in two steps. First, the data were normalized using the number of bacterial species that has an ortholog to the corresponding *S. cerevisiae* gene. This bacterial species count varies among the 1491 *S. cerevisiae* genes. In cases where the counts are different for the two genes in a pair, the normalization process should reflect the observation that the degree of co-presence/co-absence by two genes in bacterial genomes, also known as phylogenetic profile similarity, is a good indicator of functional relatedness between two bacterial genes [23]. Normalization with the lower bacterial species count would discard this useful information, because only those bacterial species with orthologs to both *S. cerevisiae* genes were considered. The higher count is therefore chosen for the normalization.

The data was then subjected to a second step of processing. Even though redundancy in the set of bacterial species has been reduced, as discussed earlier in the gene orthology prediction step, it is still possible that a gene pair can co-occur, potentially in the same operon, in a small number of related bacterial species. It has been reported that such situations adversely affect comparative-genomics-based analysis of functional relatedness between bacterial genes [21]. This problem was therefore alleviated by eliminating those gene pairs in which the higher count of bacterial species with orthologs is less than 50.

The remaining *S. cerevisiae* gene pairs were considered to be functionally related (see Results). Genes that are highly functionally related tend to have their operons conserved across bacterial genomes [15], [16] and display similar phylogenetic profile [21]. Therefore, the higher the normalized co-operon frequency, the more functionally related they are considered to be.

### C. Micro-array data and data processing

Three *S. cerevisiae* datasets for parallel measurement of transcription rate and steady-state mRNA abundance were used. The three physiological processes studied are glucose-galactose carbon source shift [3], oxidative stress [17] and osmotic stress [18], respectively. The first two datasets were downloaded from the researcher's website (http://scsie.uv.es/chipsdna/chipsdna-e.html#datos). The last one was obtained through personal communication with the authors. All are time-series data with 6 time points.

Each dataset was independently processed. Briefly, they were first transformed into folds of change relative to the first time point, and then subjected to log transformation. Log transformed data for each time point was found to be normally distributed. Finally, the log-transformed data were normalized so that the mean of all the genes, for each time point, would be zero and the standard deviation one. This was done by finding the Z-scores ((x-μ)/σ). Genes displaying flat patterns across all the time points were removed by dropping those profiles that had a standard deviation less than 0.50. Resultant data was used in our analysis to compare two genes' profiles in transcription rate and mRNA steady-state analysis.

## Results

### A. Transcription rate and mRNA abundance profile similarity between genes are bi-modally distributed

**Figure 1:**
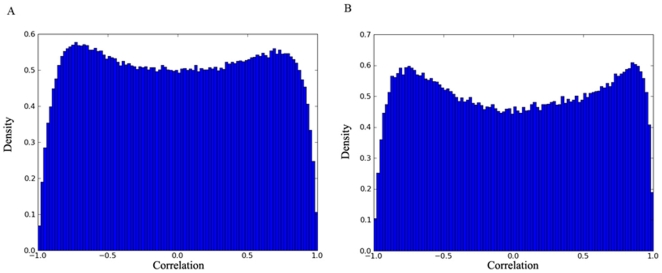
Bimodal distribution of transcription rate and mRNA abundance profile similarity. A) Distribution of correlations for mRNA abundance profiles under oxidative stress is shown; and B) distribution of correlations for transcription rate profiles under oxidative stress.

The Pearson correlation coefficient was used to measure similarity between two genes' profiles. For every condition, the set of correlations between all genes in the respective dataset was computed. Two correlations were computed for each gene pair in each of the three conditions: one between their transcription rate profiles and one between their steady-state mRNA abundance profiles. The distribution of the resulting correlations was examined to determine a strategy for further analysis. None of the correlations is normally distributed. Instead, both correlations in all three conditions, displayed bi-modal distribution, with peaks at high negative and positive correlation. Figure 1 displays the distribution of steady-state mRNA abundance correlation (panel A) and transcription rate correlations (panel B) in oxidative stress condition.

**Figure 2:**
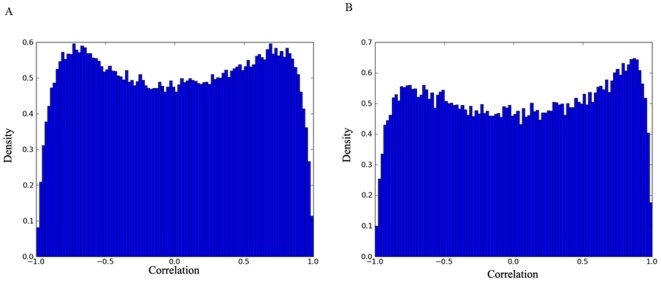
Bimodal distribution of transcription rate and mRNA abundance profile similarity between *S. cerevisiae* genes with bacterial orthologs. Distribution of correlations for mRNA abundance (A), as well as the distribution of correlations for corresponding transcription rate profiles (B), under oxidative stress are shown.

The distribution of correlations was also examined for the subset of *S. cerevisiae* genes that have bacterial orthologs to see whether they would be significantly different from the bi-modal distribution discussed above. They were all found to be bimodal, with peaks at high negative and positive correlations. Figure 2 displays the distribution of steady-state mRNA abundance correlation (panel A) for genes with bacterial ortholgs and the corresponding transcription rate correlations (panel B) in oxidative stress condition. These bimodal distributions, with two peaks not significantly different from each other in their sizes, serve as a good background model for our analysis. When analyzing only those *S. cerevisiae* gene pairs whose bacterial orthologs co-occur in the same operons, the mass of the distribution should shift to the right, enlarging the positive correlation peak while reducing the negative correlation peak (see Results section D).

### B. The bimodal distribution was explained by simulation analysis

**Figure 3:**
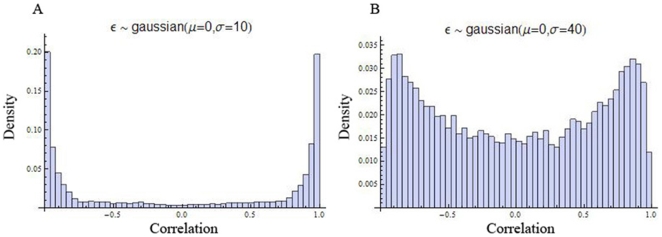
Explanation of the bi-modal distribution by simulation analysis. Distribution of correlations generated from simulations with σ = 10 (A) and 40 (B) are shown.

To investigate why the background distributions discussed above are bimodal, a simulation was performed. The simulation started with a simplistic assumption that all gene profiles consist of linear increases or decreases and could be modeled with a linear function of time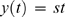. Gene profiles were generated randomly, each with a random slope, s, drawn from a uniform distribution from the interval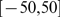. Values for time points were computed directly from the line equation using consecutive values for t. In such a model, every gene profile is either increasing or decreasing, so all correlations are necessarily either −1 or 1. Not all profiles in a micro-array time-series datasets are mono-tone; many fluctuate. To account for this observation and the intrinsic noise in a micro-array experiment, an error term was added to every time point. The error was modeled by a Gaussian distribution with parameters μ = 0 and σ. As the noise (σ) was increased, the resulting distributions came to resemble the distributions obtained from experimental data (Figure 3). When the noise is small relative to the signal (the range of the slope), there are peaks in the histogram at -1 and 1 and the probability mass decreases monotonically in both directions toward 0 (Figure 3A). As the noise is increased relative to the signal, the peaks shift toward zero in both directions (Figure 3B). Eventually, the distribution resembled a Gaussian distribution, where only one peak can be identified (Data not shown).

### C. Discrepancy between transcription rate and mRNA steady-state abundance

Steady-state mRNA abundance is determined, as mentioned earlier, by both transcription rate and mRNA degradation rate. Thus, steady-state mRNA abundance does not always correlate well with transcription rate. For example, ∼41% of *S. cerevisiae* genes exhibit lower than 0.5 correlation coefficients between their mRNA abundance and transcription rate profiles in the glucose-to-galactose shift experiment; ∼8% exhibit significant negative correlation [3].

**Figure 4:**
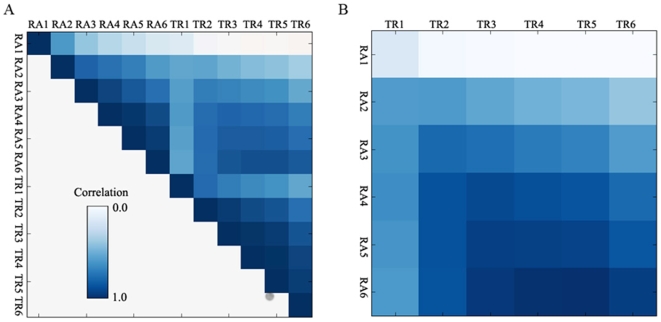
A time-delay effect from transcription rate data to mRNA abundance data. A) Correlation matrix between time points for mRNA abundance (RA) and transcription rate (TR) analysis under an osmotic stress condition is shown; B) Only correlations between TR and RA shown.

Another type of discrepancy, a time-delay effect, was observed in this study (Figure 4). Shown in figure 4A is a correlation matrix of the datasets from the osmotic stress experiment. The datasets contain 6 columns (time points) for transcription rate (TR) analysis and 6 columns (time points) for steady state mRNA abundance (RA) analysis; a column contains a reading for each gene at the corresponding time point and analysis. The correlation matrix display pair-wise Pearson correlation coefficients among the 12 columns. Each column correlates perfectly with itself as shown by the diagonal in figure 4A. Part of the matrix that correlates TR columns with RA columns was enlarged in figure 4B. TR columns display better forward correlation. The 1st TR time point (TR1) correlated better with the 2nd RA time point (RA2) than with the 1st RA time point (RA1). Similarly, The 2nd TR time point (TR2) correlated better with the 3rd RA time point (RA3) than with the 2nd RA time point (RA2); the 3rd TR time point (TR3) correlated better with the 4th RA time point (RA4) than with the 3rd RA time point (RA3); and etc. Consistently, the RA column correlated better backward; the 6^th^ RA time point (RA6) correlated better with the 5^th^ TR time point (TR5) than with the 6^th^ TR time point (TR6). Thus, a time-delay effect, as our analysis suggested, exists from TR to RNA.

### D. Transcription rate correlates better with functional relatedness

Transcription takes time. The delay effect discussed above was therefore biologically meaningful. Moreover, it confirms the quality of the datasets and the way they are processed in this study. Next, we examined whether transcription rate or steady-state mRNA abundance is a better measurement of functional relatedness between two genes.

#### D.1. Co-occurrence in the same operon by bacterial orthologs indicates functional relatedness between *S. cerevisiae* genes

**Figure 5:**
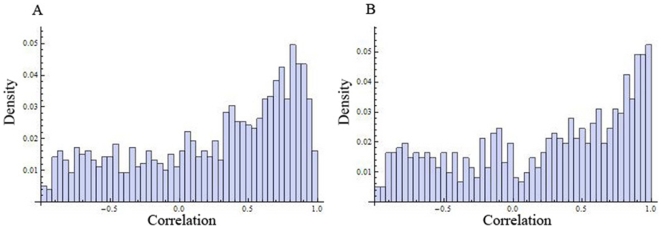
Co-occurrence in the same operon by their bacterial orthologs indicates functional relatedness between *S. cerevisiae* genes. Distribution of correlations between genes whose bacterial orthologs co-occur in the same operon at least once are shown; A) data for mRNA abundance levels; B) data for transcription rates.

Co-occurrence in the same operon (co-operon) by the two genes in bacterial genomes indicates functional relatedness; such genes often participate in the same biochemical pathways [24]. We tested whether two *S. cerevisiae* genes are functionally related when their bacterial orthologs putatively co-occur in the same operon (see Materials and Methods). Figure 5 displays the distributions of pair-wise correlation coefficients for yeast gene pairs whose bacterial orthologs display a co-operon frequency of one or more. When compared with the distributions in figures 1 and 2, significant positive correlation coefficients were substantially enriched. As discussed earlier (see Results section A), the mass of the distribution shifted to the right, dramatically enlarging the positive correlation peak. Significant negative correlations were substantially reduced; the peaks corresponding to negative correlation coefficient were severely reduced when compared with the corresponding peaks in figures 1 and 2. Thus, having their bacterial orthologs sharing an operon strongly indicated functional relatedness between two *S. cerevisiae* genes.

#### D.2. Transcription rate correlates better with co-operon frequency

**Figure 6:**
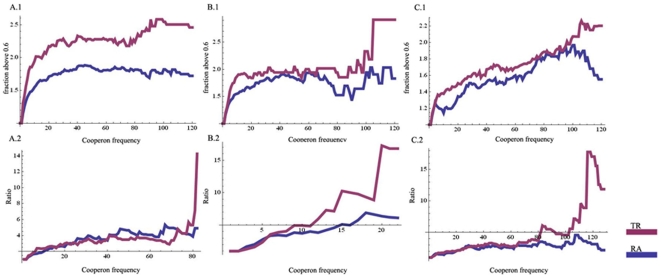
Tanscription rate data correlates better with co-operon frequency. Relationship between transcription rate correlation (TR), as well as mRNA abundance level correlation (RA), and co-operon frequency for *S. cerevisiae* gene pairs whose bacterial orthologs co-occur in the same operons are shown. For each column, the top plot represents the fraction of points above 0.6 as cooperon frequency increases; the bottom plot shows the ratio between the number of points above 0.6 and below −0.6 with increasing cooperon frequency. The bottom plot ends at cooperon frequency where the number of point below −0.6 becomes 0 in the TR data, as the cooperon frequency where the number of point below −0.6 becomes 0 is always higher in the RA data. Both plots are given relative (folds of change) to the first time point. Column A) gives plots for galactose-glucose shift data; column B) gives plots for oxidative stress data; column C) gives plots for osmotic stress data.

In order to compare transcription rate and mRNA abundance as measurement of functional relatedness, we need an independent measurement of functional relatedness to compare the two factors to. Operons containing highly functionally related genes are, as revealed by comparative genomic analysis of bacterial genomes, more likely to be conserved across species [15], [16]. The more an operon is conserved, the more functionally related its genes are. Indeed, the winning method in a comparison of 30 operon prediction methods utilizes cross species conservation as a major predictive component [25]. Therefore, we used normalized co-operon frequency by their bacterial orthologs, as discussed in Materials and Methods, as a measurement of functional relatedness between two *S. cerevisiae* genes. For all such gene pairs, we already have two correlation coefficients: one between their transcription rate profiles and one between their mRNA abundance profiles. We tested which one of the two correlation coefficients correlate better with the corresponding co-operon frequencies as we increase the minimum co-operon frequency requirement. Two approaches were used based on the observation that distributions of the correlation coefficients are bimodal (see Results section A). We calculated the fraction of the correlation coefficients that are equal or bigger than 0.6, as well as the fraction of the correlation coefficients that are equal or smaller than −0.6. In the first approach, we examined how the fraction of those correlation coefficients, which are equal to or bigger than 0.6, changed along with the elevating co-operon frequency (Fig. 6, top half). In the second approach, we calculated the ratio of the two fractions and examined how the ratio changed along with the elevating co-operon frequency (Fig. 6, bottom half). Across all three experimental conditions and in both approaches, transcription rate data displayed better positive relationship with elevating co-operon frequency than mRNA abundance data. Transcription rate, thus, seems a better indicator of functional relatedness.

## Discussion

Understanding cellular logic in gene expression regulation is crucial for network and systems biology. Biochemical network construction can, to some degree, be considered a reverse engineering process - inference of the structure of the networks from their dynamic behavior [26], which is often measured in terms of gene expression levels. Using the right logic/assumption is a prerequisite for such efforts to be successful. Once established, a biochemical network model is useful only when theoretical prediction can be made out of it. Once again, using the right logic is the key to any accurate prediction.

Gene expression analysis is, due to its prominent role in cellular dynamics, a critical component of network and systems biology [4]. At the mRNA level, some measurement technologies are hybridization based. Some are sequencing based, measuring mRNA levels as the numbers of times a gene's tags occur in the sequence set [8], [9]. These measurement technologies are less expensive and more developed than those at the protein level. Therefore, gene expression analysis is done, so far, more frequently at the mRNA levels. Most efforts measure steady-state mRNA abundance. Our analysis suggests such datasets should be approached with caution; it is important not to over interpret such data in network analysis and regulatory sequence analysis.

While undoubtedly informative, steady-state mRNA abundance data analysis is complicated by the fact that mRNA abundance is determined by two factors: transcription rate and mRNA degradation rate. It is imperative to keep in mind that the discrepancy between transcriptional rate and steady-state mRNA abundance is frequently observed; a gene's steady-state abundance profile during a physiological process may not be explained by its transcription rate profile. For example, the cells store mRNA molecules in the EGP bodies and the p-bodies (processing bodies) [27], [28], so these mRNAs remain abundant even though no new molecules are being produced. However, the precise cellular logic involved in transcription and mRNA degradation regulation remain to be un-covered.

At the transcription rate level, an important factor might be to optimally allocate metabolic resources among the set of biochemical pathways, and at the same time, meet the demands of biochemical network dynamics. Gene expression carries expensive metabolic overhead. Signaling pathways are often used to regulate transcription factors to specify the genes for active transcription. A signaling step, usually a protein kinase, consumes an ATP molecule for protein phosphorylation. Upon initiation, the transcribing RNA polymerase consumes more ATP molecules and ribo-nucleotide building blocks to synthesize transcript product of the gene. Thus it is vital to utilize the signaling and the transcription machineries in the most economical manner, and to minimize un-necessary wasteful transcription events.

For transcription regulation, genomic regulatory sequences play prominent roles in transcription regulation. Important signals embedded in the sequences include short motifs, to which transcription factors bind to activate or turn off transcription. Even though eukaryotic genes are independent transcriptional units, functionally related genes tend to have similar expression profiles. It is a general assumption that regulatory sequences of such genes embed similar profiles of transcription factor binding sites, often termed transcription factor binding site modules. Computational analysis of eukaryotic regulatory sequences, however, remains technically challenging; satisfactory predictive power remains elusive. Part of the blame might be low signal-to-noise ratio, as transcription factor binding sites are generally short and permutation tolerant. It might also be partially due to the fact that we have not understood eukaryotic transcription regulation well enough yet. Additionally, our finding suggests that using steady-state mRNA abundance data as input to such computational analysis, as is generally practiced, might bear part of the blame as well. Transcription rate data, which is free from interference by mRNA degradation, should be a better input in regulatory sequence analysis.

While our understanding of transcription regulation is far from satisfactory, we understand even less about mRNA degradation regulation. Much progress has been made, especially the recent discovery of miRNA mediated mRNA degradation mechanism [29]. Functionally related genes tend to have their mRNA products co-degraded, thus the term “degradation regulon” has been used to describe this phenomenon. But much of the cellular logic in mRNA degradation regulation remains to be deciphered. Datasets that can be used to systematically correlate degradation profiles and functional relatedness for gene pairs, as was done at transcription rate level in this study, remain to be produced. Additionally, relationship between transcription regulons and degradation regulons, i.e., how much they overlap, remains to be explored.

Similar issues exist for analyzing gene expression regulation logic at the protein level. Current efforts generally measure steady-state protein abundance. Steady-state levels of proteins, similar to that of mRNAs, are determined by two factors: translation rate and degradation rate. While biologically informative, steady-state abundance data might not be the best to use to decipher the cellular logic in the dynamic behavior of biochemical networks. Translation is a metabolically expensive process: it uses ATP molecules as energy supply and amino acids as building blocks. Optimal allocation of these resources among the collection of biochemical pathways should be an important part of biochemical network dynamics.

Additionally, gene expression profiling has also been widely used in clinical research. Currently, disease diagnosis is primarily histology and morphology based. Therapy is generic for all patients. The goal of genomic and personalized medicine is to improve disease diagnosis at molecular level and personalized therapy tailored for individual patients. Potential usage of gene expression profiling in these area is under intensive investigation. For instance, gene expression profiling has been explored as a potential tool to improve the accuracy of tumor classification and prognosis [30], which so far are largely histology and morphology based. Our finding suggests that revising the protocol to incorporate additional information such as transcription rate data, for which genome wide analysis technology is readily available, deserves further exploration.

## References

[1] Barabasi AL, Oltvai ZN (2004). Network biology: understanding the cell's functional organization.. Nature reviews.

[2] Davidson EH, Rast JP, Oliveri P, Ransick A, Calestani C (2002). A Genomic Regulatory Network for Development.. Science.

[3] Garcia-Martinez J, Aranda A, Perez-Ortin JE (2004). Genomic run-on evaluates transcription rates for all yeast genes and identifies gene regulatory mechanisms.. Molecular cell.

[4] Ideker T, Thorsson V, Ranish JA, Christmas R, Buhler J (2001). Integrated genomic and proteomic analyses of a systematically perturbed metabolic network.. Science.

[5] Hunter T (2009). Tyrosine phosphorylation: thirty years and counting.. Current opinion in cell biology.

[6] Tissot B, North SJ, Ceroni A, Pang PC, Panico M (2009). Glycoproteomics: past, present and future.. FEBS letters.

[7] Laskowski RA, Gerick F, Thornton JM (2009). The structural basis of allosteric regulation in proteins.. FEBS letters.

[8] Anisimov SV (2008). Serial Analysis of Gene Expression (SAGE): 13 years of application in research.. Current pharmaceutical biotechnology.

[9] Teng X, Xiao H (2009). Perspectives of DNA microarray and next-generation DNA sequencing technologies.. Science in China.

[10] Guan Y, Myers CL, Lu R, Lemischka IR, Bult CJ (2008). A genomewide functional network for the laboratory mouse.. PLoS computational biology.

[11] Beer MA, Tavazoie S (2004). Predicting gene expression from sequence.. Cell.

[12] Yuan Y, Guo L, Shen L, Liu JS (2007). Predicting Gene Expression from Sequence: A Reexamination.. PLoS computational biology.

[13] Manson McGuire A, Church GM (2000). Predicting regulons and their cis-regulatory motifs by comparative genomics.. Nucleic acids research.

[14] Blattner FR, Plunkett G, Bloch CA, Perna NT, Burland V (1997). The complete genome sequence of Escherichia coli K-12.. Science.

[15] Dam P, Olman V, Harris K, Su Z, Xu Y (2007). Operon prediction using both genome-specific and general genomic information.. Nucleic acids research.

[16] Wu H, Mao F, Su Z, Olman V, Xu Y (2005). Prediction of functional modules based on gene distributions in microbial genomes.. Genome informatics.

[17] Molina-Navarro MM, Castells-Roca L, Belli G, Garcia-Martinez J, Marin-Navarro J (2008). Comprehensive transcriptional analysis of the oxidative response in yeast.. J Biol Chem.

[18] Romero-Santacreu L, Moreno J, Perez-Ortin JE, Alepuz P (2009). Specific and global regulation of mRNA stability during osmotic stress in Saccharomyces cerevisiae.. RNA (New York, N.Y.

[19] Altschul SF, Madden T L, Schaffer AA, Zhang J, Zhang Z (1997). Gapped BLAST and PSI-BLAST: a new generation of protein database search programs.. Nucleic acids research.

[20] Karimpour-Fard A, Hunter L, Gill RT (2007). Investigation of factors affecting prediction of protein-protein interaction networks by phylogenetic profiling.. BMC genomics.

[21] Moreno-Hagelsieb G, Janga SC (2008). Operons and the effect of genome redundancy in deciphering functional relationships using phylogenetic profiles.. Proteins.

[22] Yellaboina S, Goyal K, Mande SC (2007). Inferring genome-wide functional linkages in E. coli by combining improved genome context methods: comparison with high-throughput experimental data.. Genome research.

[23] Pellegrini M, Marcotte EM, Thompson MJ, Eisenberg D, Yeates TO (1999). Assigning protein functions by comparative genome analysis: protein phylogenetic profiles.. Proceedings of the National Academy of Sciences of the United States of America.

[24] Mao F, Dam P, Chou J, Olman V, Xu Y (2009). DOOR: a database for prokaryotic operons.. Nucleic acids research.

[25] Brouwer RW, Kuipers OP, van Hijum SA (2008). The relative value of operon predictions.. Briefings in bioinformatics.

[26] Kitano H (2002). Systems Biology: A Brief Overview.. Science.

[27] Franks TM, Lykke-Andersen J (2008). The control of mRNA decapping and P-body formation.. Molecular cell.

[28] Hoyle NP, Castelli LM, Campbell SG, Holmes LE, Ashe MP (2007). Stress-dependent relocalization of translationally primed mRNPs to cytoplasmic granules that are kinetically and spatially distinct from P-bodies.. The Journal of cell biology.

[29] Chua JH, Armugam A, Jeyaseelan K (2009). MicroRNAs: biogenesis, function and applications.. Current opinion in molecular therapeutics.

[30] Marchionni L, Wilson RF, Marinopoulos SS, Wolff AC, Parmigiani G (2007). Impact of gene expression profiling tests on breast cancer outcomes.. Evidence report/technology assessment.

